# Id Proteins Suppress E2A-Driven Invariant Natural Killer T Cell Development prior to TCR Selection

**DOI:** 10.3389/fimmu.2018.00042

**Published:** 2018-01-24

**Authors:** Sumedha Roy, Amanda J. Moore, Cassandra Love, Anupama Reddy, Deepthi Rajagopalan, Sandeep S. Dave, Leping Li, Cornelis Murre, Yuan Zhuang

**Affiliations:** ^1^Department of Immunology, Duke University Medical Center, Durham, NC, United States; ^2^Department of Molecular Biology, University of California, San Diego, La Jolla, CA, United States; ^3^Duke Institute for Genome Sciences and Policy, Duke University, Durham, NC, United States; ^4^Biostatistics and Computational Biology Laboratory, National Institute of Environmental Health Sciences, National Institutes of Health (NIH), Durham, NC, United States

**Keywords:** natural killer T cells, innate lymphocytes, Id proteins, E2A, thymus

## Abstract

A family of transcription factors known as E proteins, and their antagonists, Id proteins, regulate T cell differentiation at critical developmental checkpoints. Id proteins promote the differentiation of conventional αβ T cells and suppress the expansion of innate-like αβ T cells known as invariant natural killer T (iNKT) cells. However, it remains to be determined whether Id proteins differentially regulate these distinct lineage choices in early stages of T cell development. In this manuscript, we report that in Id-deficient mice, uninhibited activity of the E protein family member E2A mediates activation of genes that support iNKT cell development and function. There is also biased rearrangement in Id-deficient DP cells that promotes selection into the iNKT lineage in these mice. The observed expansion of iNKT cells is not abrogated by blocking pre-TCR signaling, which is required for conventional αβ T cell development. Finally, E2A is found to be a key transcriptional regulator of both iNKT and γδNKT lineages, which appear to have shared lineage history. Therefore, our study reveals a previously unappreciated role of E2A in coordinating the development of the iNKT lineage at an early stage, prior to their TCR-mediated selection alongside conventional αβ T cells.

## Introduction

The thymic output of a diverse and abundant population of conventional CD4^+^ and CD8^+^ αβ T cells constitutes the adaptive immune system that is necessary for a specific and effective immune response to antigens. A smaller but significant population of unconventional T cells concomitantly develops in the thymus, with innate-like capabilities of mounting a rapid and potent immune response (1). These innate-like T cells have garnered increasing interest as their memory phenotype can be harnessed in the context of allergies, infections, and tumors. Innate-like T cell populations include TCRαβ^+^ natural killer T (NKT) cells, TCRγδ^+^ NKT cells, innate-like CD8^+^ T cells, CD8αα intraepithelial lymphocytes, and mucosal-associated invariant T cells. Invariant NKT (iNKT) cells are among the best characterized innate-like T cells, which arise in parallel with conventional αβ T cells. These cells are thought to stochastically express a canonical Vα14-Jα18 TCRα chain at the CD4^+^CD8^+^ double positive (DP) stage, which allows them to undergo TCR selection mediated by a CD1d molecule expressed on other conventional DP thymocytes (2, 3). γδNKT cells are yet another population of innate-like γδ T cells that express a restricted Vγ1.1Vδ6.3 TCR (4). Both iNKT and γδNKT cells are characterized by high levels of expression of the innate-like transcription factor, promyelocytic zinc finger (PLZF), and readily produce effector cytokines like IL-4 (5, 6). While the transcriptional programs that drive conventional CD4^+^ and CD8^+^ T cell specification and development have been well characterized, little is known about the innate-specific transcriptional programs upstream of PLZF that are responsible for the divergence of innate-like T cells from conventional T cells (7).

Id proteins, primarily produced by *Id2* and *Id3* during T cell development, are inhibitors of the E protein transcription factors E2A and HEB (8, 9). Interestingly, Id proteins play opposite roles in the development of conventional and innate-like T cells, such that they promote the former and suppress the latter. In response to pre-TCR and TCR signals, inhibition of E protein activity by Id proteins plays a critical role in promoting the differentiation and positive selection of conventional αβ T cells, such that disruption of *Id2* and *Id3* impairs conventional αβ T cell development beyond the TCR checkpoint (10). Analogous to αβ T cell development, the function of Id3 in promoting conventional γδ T cell development has also been mapped downstream of the γδ TCR (11). In contrast, large populations of iNKT, γδNKT, and innate variant T_FH_ cells have been observed in the same Id3- and Id2/Id3-deficient animals, indicating a negative role for Id proteins in regulating innate-like T cell development (12–17). However, the mechanism that drives the development and expansion of these innate-like T cell populations in Id-deficient mice is still elusive. Given the reciprocal nature of Id proteins in supporting conventional T cells and suppressing innate-like T cells, it is reasonable to predict that Id proteins control innate-like T cell development through a somewhat distinct mechanism from conventional T cells. Interestingly, Id proteins have been shown to modulate E protein activity during early stages of T cell development (8). Therefore, it remains to be determined whether Id-mediated suppression of these innate-like T cells is limited to cell expansion after selection and lineage commitment, or if it also influences their lineage choice at earlier stages of development.

In this manuscript, we report biased Vα14-Jα18 rearrangements and E2A-driven regulation of genes that promote the iNKT lineage in DP cells of Id-deficient mice. Further, a block in pre-TCR signaling hinders conventional αβ T cell development but fails to eliminate the expanded innate-like iNKT and γδNKT cells in Id-deficient mice. Our study reveals a distinct regulatory event that separates iNKT cell lineage from the conventional αβ T cell lineage prior to the TCR signal. Additionally, we define an E2A-mediated transcription network that supports innate-like iNKT and γδNKT lineages.

## Results

### Absence of Id Proteins Allows E2A to Induce Genes Involved in iNKT Cell Development and Function

**Figure 1:**
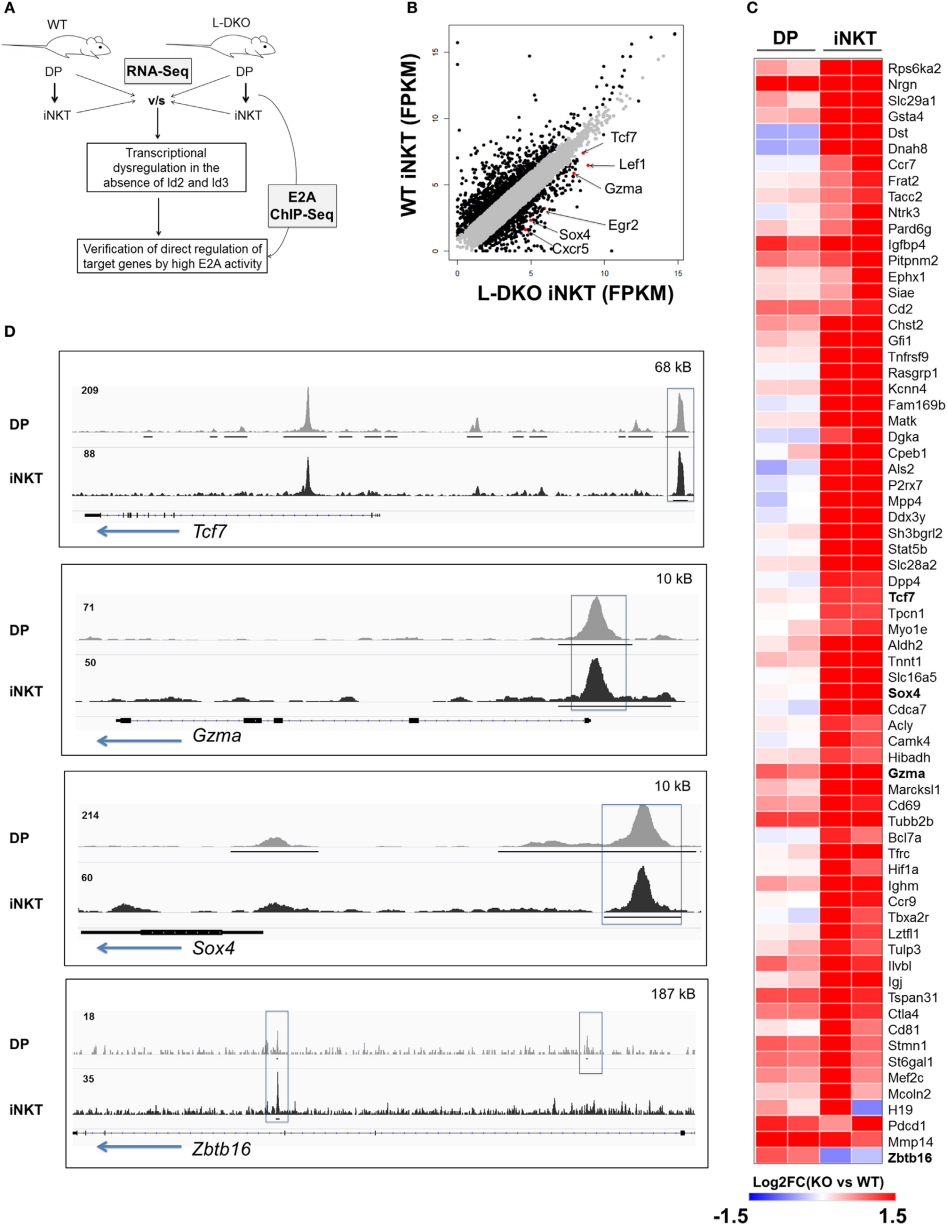
E2A drives transcriptional programs that support iNKT cell development and function in the absence of Id proteins. **(A)** Schematic showing RNA-Seq and ChIP-Seq analysis depicted in **(B–D)**. **(B)** Gene expression (FPKM, fragments per kilobase exon-model per million reads mapped) in wild-type (WT) and L-DKO iNKT cells with key genes highlighted (red). Genes with greater than twofold difference in L-DKO or WT iNKT cells are marked in black and rest in gray. **(C)** Gene expression patterns in L-DKO DP and L-DKO iNKT cells (fold change over corresponding WT cells) for genes that are more than twofold upregulated in L-DKO iNKT cells compared to WT iNKT cells and are part of the iNKT transcriptional program (Msigdb ID: M18517) or inflammatory responses (Msigdb ID: M5913). **(D)** E2A ChIP-Seq peaks in L-DKO DP and L-DKO iNKT samples. Solid black lines underneath the tracks indicate significant (*p*-value less than 10^−5^) peaks called by MACS. Important E2A peaks are highlighted by boxes within each panel. Length of the genome in each panel is indicated on the top right. Numbers at the top of each track indicate the maximum peak height.

Our laboratory and others have shown that the loss of function of Id3 or Id2/Id3 results in a significant increase in numbers of iNKT cells (12, 17–20). We hypothesized that uninhibited E2A activity in the absence of Id proteins may induce genes important for the iNKT developmental program. Therefore, we sought to identify specific downstream gene targets that drive the expansion of iNKT cells in Id2/Id3-deficient mice (Id2^f/f^Id3^f/f^LckCre^+^, LckCre-mediated double knockout or L-DKO) by performing RNA-Seq and E2A ChIP-Seq analysis in L-DKO DP and L-DKO iNKT cells, as representative populations prior to, and after CD1d-mediated selection (Figure 1A). Comparing the transcription profile of L-DKO iNKT cells to wild type (WT) iNKT cells, we found 552 genes to be upregulated by more than twofold in L-DKO iNKT cells with respect to WT iNKT cells (Figure 1B). Pathway analysis confirmed significant upregulation of genes related to iNKT differentiation and effector function (Figure S1A in Supplementary Material; Figure 1C). Genes essential for iNKT development and function, such as *Tcf7, Sox4*, and *Gzma*, were highly upregulated in iNKT cells deficient in Id proteins (21, 22). A subset of genes upregulated in L-DKO iNKT cells were also upregulated in L-DKO DP cells compared to WT DP cells (Figure 1C). *Zbtb16*, which is highly expressed in WT iNKT cells, was found to be prematurely activated in L-DKO DP cells (5). ChIP-Seq analysis of L-DKO DP and iNKT cells further verified strong E2A binding to the promoter and/or enhancer regions of the highly upregulated genes (Figure 1D), indicating a direct role for E2A in initiating and/or maintaining the transcription of these target genes. Overall, these findings suggested E2A-mediated promotion of iNKT cell development in the absence of Id proteins.

### E2A Supports iNKT Cell Fate at the DP Stage through Activation and Collaboration with Relevant Downstream Transcription Factors

**Figure 2:**
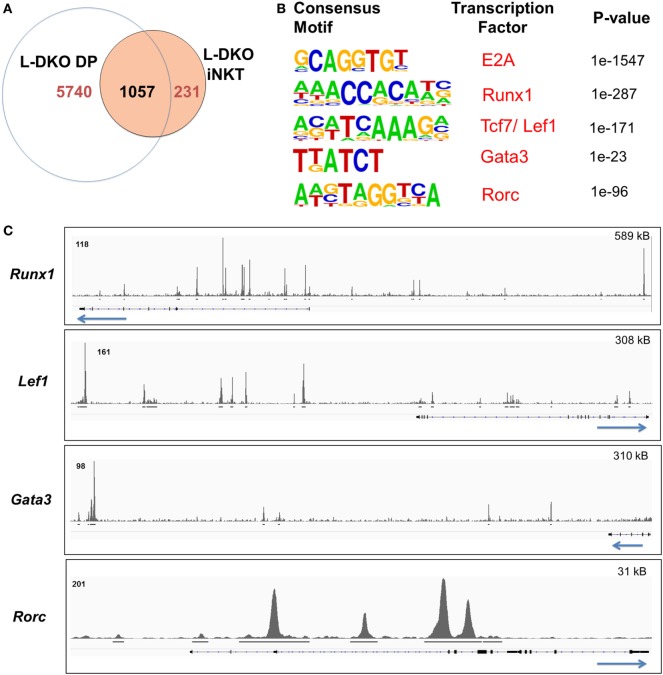
E2A regulates and collaborates with downstream transcription factors to promote the iNKT lineage fate choice at the DP stage. **(A)** Number of genes with E2A binding in L-DKO DP and/or L-DKO iNKT cells. **(B)** *De novo* motif analysis in L-DKO DP cells, with predicted consensus motifs within E2A peaks, corresponding transcription factors, and *p* values. **(C)** E2A peaks in L-DKO DP cells at loci for motifs identified in **(B)**. Solid black lines underneath each track indicate significant (*p*-value less than 10^−5^) peaks called by MACS. Length of the genome depicted in each panel is indicated on the top right. Numbers at the top of each track indicate the maximum peak height.

We noted that Id-deficient DP cells seemed to upregulate a handful of genes related to the iNKT lineage, and many of the E2A targets in L-DKO iNKT cells were also occupied by E2A in DP cells (Figures 1C,D and 2A). The overall number of peaks in L-DKO DP cells was also much greater as compared to iNKT cells (Figure 2A). It is possible that the shared downstream targets are important for driving and sustaining iNKT fate during and after TCR selection. The choice of conventional CD4^+^ and CD8^+^ T cell fate upon TCR selection is determined by the lineage-specific transcription factors ThPOK and RUNX3, respectively (23). Along similar lines, we decided to examine involvement of transcription factors that might cooperate with E2A in differentially promoting iNKT lineage fate choice in DP cells.

We used *de novo* motif analysis to predict transcription factors that can bind to regulatory regions of identified ChIP-Seq gene targets. Besides the expected binding by E2A, this analysis demonstrated enrichment for RUNX1, TCF7, LEF1, GATA3, and RORγt motifs in our peaks, deeming them as potential partners of E2A in L-DKO DP and iNKT cells (Figure 2B; Figure S1B in Supplementary Material). Furthermore, we found E2A peaks at the genes encoding these transcription factors, indicating that E2A may directly regulate and subsequently collaborate with these factors to modulate gene expression (Figure 2C). These transcription factors have been well documented to play critical roles in iNKT cell development (3, 22, 24). RUNX1 has also been recently reported to regulate PLZF kinetics and expression through the *Zbtb16* enhancer region where E2A was bound (Figure 1D) (7). These data suggest that E2A may promote iNKT lineage fate choice in DP cells by activating and collaborating with downstream transcription factors.

### Absence of Id Proteins Promotes Vα14-Jα18 Rearrangement among Preselection DP Cells Independent of CD1d-Mediated Selection

The previous observations suggested regulation of iNKT lineage fate choice at the DP stage. RORγt, which regulates survival of DP cells and, consequently, distal iNKT TCRα (Vα14-Jα18) rearrangement, was also predicted to be a co-factor of E2A in L-DKO DP cells (Figure 2B) (25). Id gene deletion has been reported to lead to prolonged RAG1 and RAG2 expression, and one possible outcome of the persistent expression is a higher frequency of secondary, distal TCRα rearrangements, including the iNKT-specific rearrangement (18). However, we wanted to determine if Id gene deletion also has a specific impact on Vα14-Jα18 rearrangement in DP cells that have not yet undergone TCR selection. We evaluated TCRα usage in L-DKO CD1d^−/−^ mice that lack iNKT cells due to *Cd1d1* and *Cd1d2* deficiency, thereby allowing us to study the impact on TCRα rearrangement independent of CD1d-mediated selection of iNKT cells (26).

**Figure 3:**
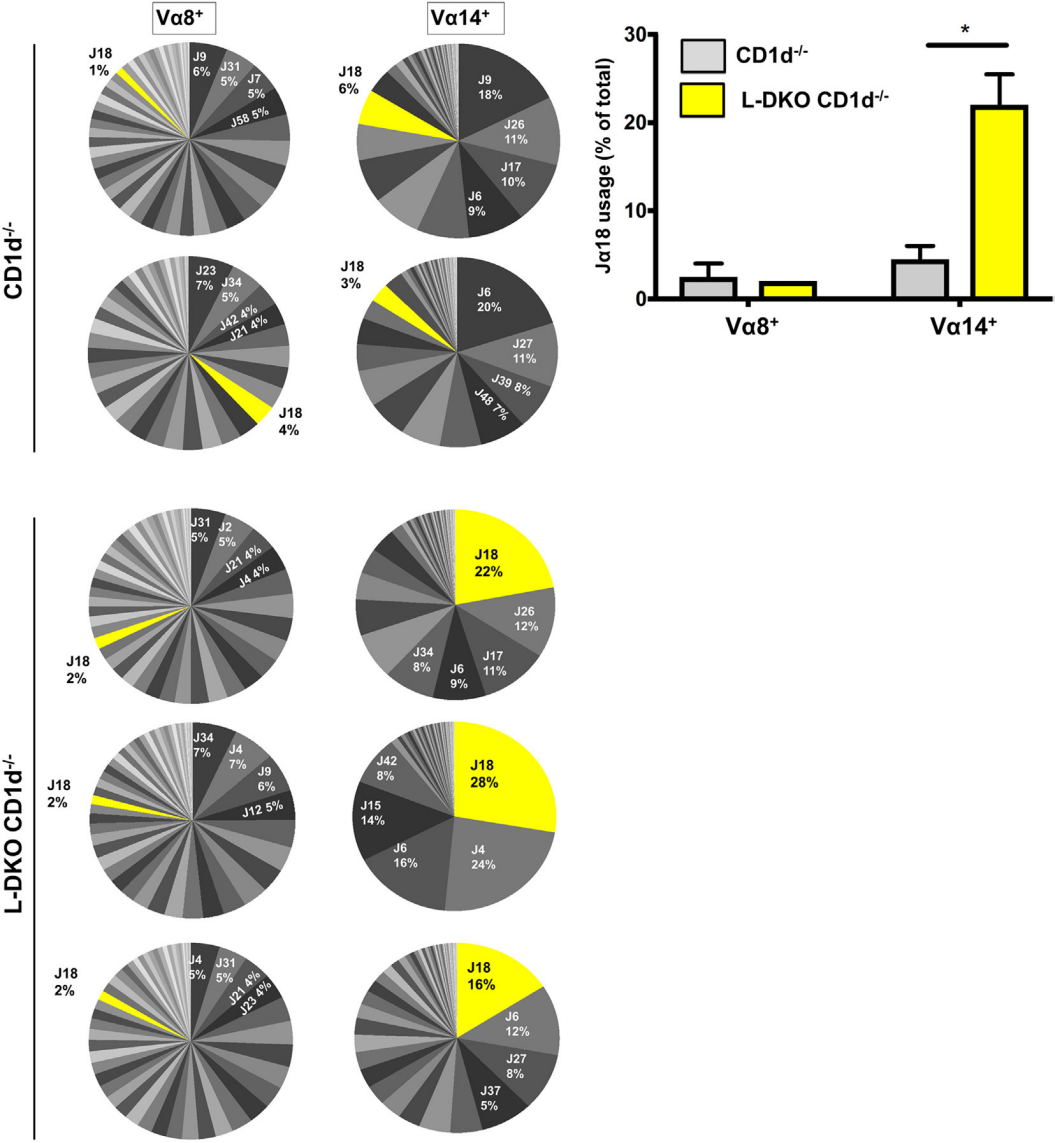
Id2/Id3 deficiency increases Vα14-Jα18 rearrangement in preselection DP pool of CD1d-deficient mice. Jα repertoire for Vα8^+^ (left panel) and Vα14^+^ (center panel) CD4^+^ CD8^+^ CD69^−^ cells in CD1d^−/−^ (*n* = 2) and L-DKO CD1d^−/−^ (*n* = 3) mice. Each pair of Vα8^+^ and Vα14^+^ pie charts represents the repertoire in a single mouse. Percentages represented by top 4 Jα chains, as well as Jα18 are indicated for each mouse. Jα chains are labeled according to new HUGO Gene Nomenclature Committee (TRAJ is abbreviated as J). Average number of total reads for CD1d^−/−^ control mice was 37,936 for Vα8^+^ cells and 27,619 for Vα14^+^ cells. For TKO mice, it was 40,925 for Vα8^+^ cells and 28,758 for Vα14^+^ cells. Bar graph (right) represents summary of Jα18 usage in CD1d^−/−^ and L-DKO CD1d^−/−^ mice. Error bars represent SEM. Statistical significance is represented by *p* values (*<0.05).

We sorted preselection DP (CD4^+^CD8^+^CD69^−^) cells from L-DKO CD1d^−/−^ and CD1d^−/−^ control mice, and sequenced Vα8^+^ and Vα14^+^ populations to compare the Jα diversity among these cells, as indicators of total preselection DP cells and potential iNKT precursors, respectively. We found no difference in the breadth of the Jα repertoire among Vα8^+^ cells in L-DKO CD1d^−/−^ or CD1d^−/−^ control mice (Figure 3). There was also no evidence to suggest increased distal Jα rearrangements in L-DKO CD1d^−/−^ mice as compared to CD1d^−/−^ mice (Figure S2A in Supplementary Material). This implied that the absence of Id proteins did not promote an overall increase in distal Jα rearrangements. However, we did find a preferential increase in the frequency of Jα18 rearrangements among Vα14^+^ cells in L-DKO CD1d^−/−^ mice as compared to CD1d^−/−^ control mice (Figure 3; Figure S2B in Supplementary Material). This increase was found in both productive and non-productive rearrangements, which verified that this outcome was not due to TCR selection (Figure S2C in Supplementary Material). Overall, this indicated that the loss of function of Id proteins causes a specific, CD1d-independent increase in the frequency of preselection DP cells that are eligible for selection into the iNKT lineage. The expression of a Vα14-Jα18 transgene can partially rescue defects in iNKT cell development and lead to an increase in iNKT cells (25, 27). Therefore, the increased bias toward iNKT-specific rearrangement in preselection DP cells is likely to contribute to the increased iNKT population in Id-deficient mice in synergy with additional E2A-mediated transcriptional programs.

### Block in Pre-TCR Signaling Does Not Diminish Expanded iNKT Cell Population

E2A-mediated regulation of iNKT-relevant downstream targets in DP cells, and an early bias in iNKT TCRα rearrangement among preselection cells in our Id-deficient mouse models prompted us to consider the possibility that Id proteins may suppress lineage specification for iNKT cells early in T cell development. Therefore, we decided to investigate if these populations are impacted by the deficiency of Id proteins at the pre-TCR checkpoint (at the DN3 stage), which regulates conventional αβ T cell development. Mice deficient in pre-Tα have restricted T cell development, with the majority of cells blocked at the DN stage (28). pTα^−/−^ mice are also known to completely lack iNKT cells (29). We generated L-DKO pTα^−/−^ mice to examine how blocking pre-TCR signaling impacted the expanded iNKT population in L-DKO mice.

**Figure 4:**
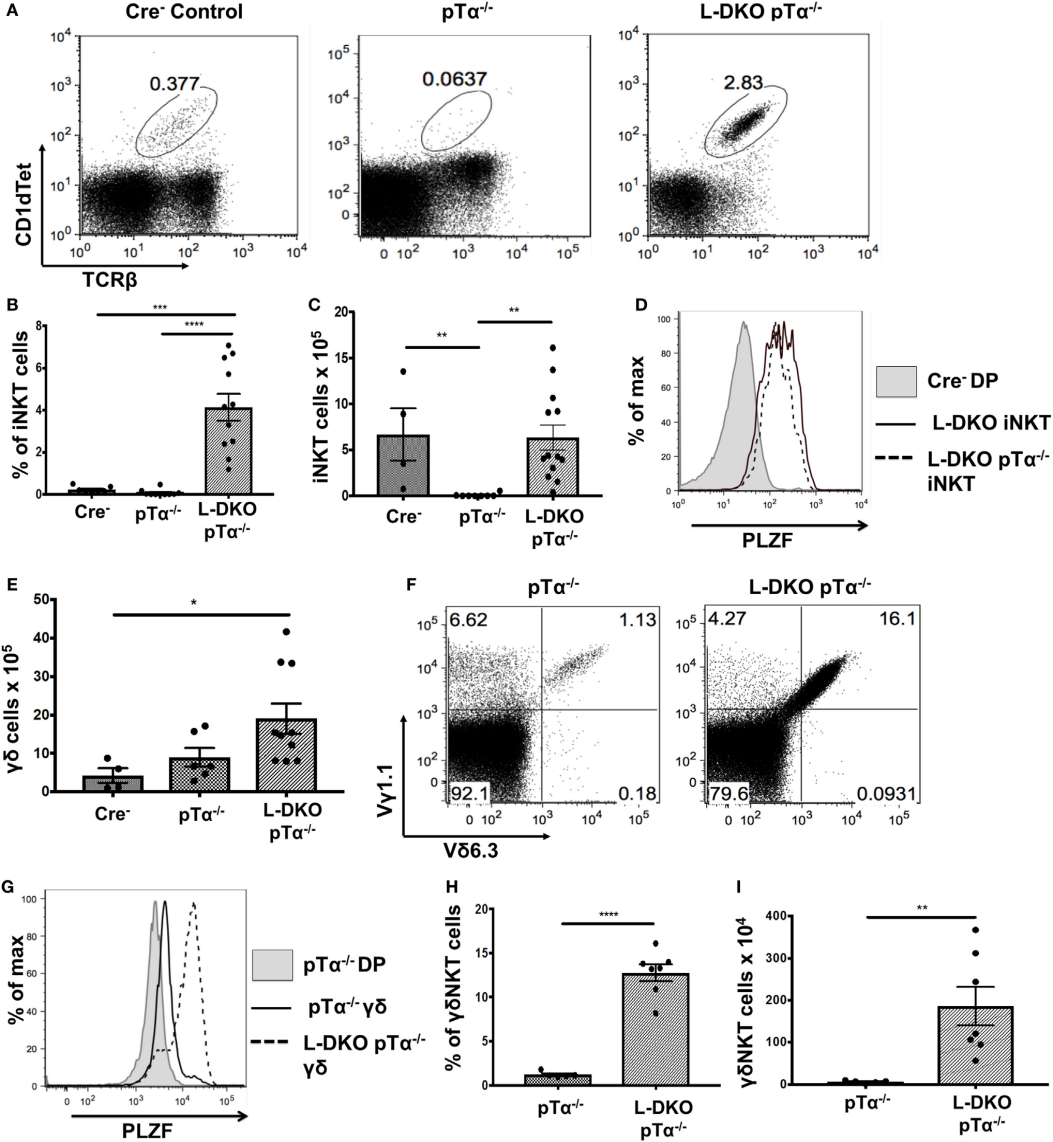
A block in pre-TCR signaling does not arrest enhanced iNKT cell and γδNKT populations in the absence of Id proteins. **(A)** Representative iNKT cells in 3- to 5-week-old Cre^–^ (*n* = 4), pTα^−/−^ (*n* = 10), and L-DKO pTα^−/−^ (*n* = 11) mice. **(B)** Percentage and **(C)** numbers of iNKT cells in 3- to 5-week-old mice. **(D)** Representative promyelocytic zinc finger (PLZF) expression levels in iNKT cells from 3- to 5-week-old Cre^−^ (*n* = 3), L-DKO (*n* = 3), and L-DKO pTα^−/−^ (*n* = 4) mice. **(E)** Total number of γδ T cells in 3- to 5-week-old Cre^−^ (*n* = 4), pTα^−/−^ (*n* = 6), L-DKO pTα^−/−^ (*n* = 10) mice. **(F)** Representative γδNKT (Vγ1.1^+^Vδ6.3^+^) populations in 3- to 5-week-old pTα^−/−^ (*n* = 5), L-DKO pTα^−/−^ (*n* = 7) mice. **(G)** Representative PLZF expression levels in γδ T cells from 3- to 5-week-old Cre^−^ (*n* = 3), L-DKO (*n* = 3), and L-DKO pTα^−/−^ (*n* = 4) mice. **(H)** Percentages and **(I)** numbers of γδNKT cells in mice shown in **(F)**. Error bars represent SEM. Statistical significance is represented by *p* values (*<0.05, **0.005, ***<0.0005, n.s. >0.05).

Despite the complete absence of iNKT cells in pTα^−/−^ mice, to our surprise, we found a robust iNKT population in L-DKO pTα^−/−^ mice (Figures 4A–C). These iNKT cells also expressed high levels of PLZF (Figure 4D). It is known that pTα^−/−^ mice have an increase in γδ T cells, and L-DKO pTα^−/−^ mice showed a similar increase in the γδ population compared to WT mice (Figure 4E) (28). However, the γδ T cells in L-DKO pTα^−/−^ mice were predominantly Vγ1.1^+^Vδ6.3^+^ and uniformly upregulated PLZF, reflecting a specific increase in innate-like γδNKT cells in these mice (Figures 4F–I).

**Figure 5:**
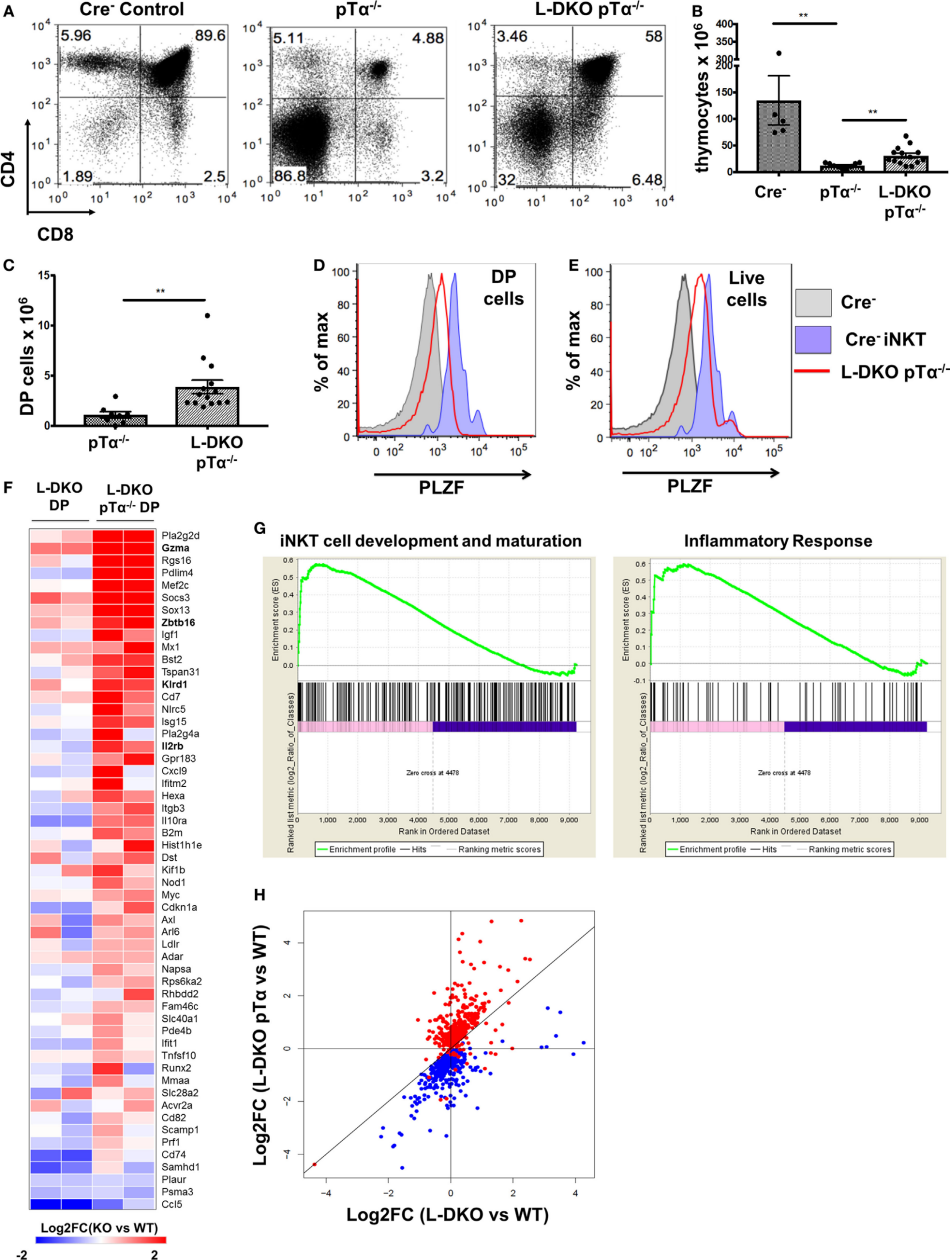
Adoption of innate-like transcriptional program by DP cells in the absence of pre-TCR signaling and Id proteins. **(A)** Representative thymocytes in 3- to 5-week-old Cre^−^, pTα^−/−^, and L-DKO pTα^−/−^ mice as shown by CD4 and CD8 staining. **(B)** Total number of thymocytes in 3- to 5-week-old mice. **(C)** Number of DP cells in mice shown in **(A)**. 5–13 mice included for each genotype shown in **(A–C)**. **(D,E)** Representative promyelocytic zinc finger (PLZF) expression levels in DP cells (CD1dTet^−^ TCRγδ^−^) from 3- to 5-week-old Cre^−^ (solid gray, *n* = 3) and L-DKO pTα^−/−^ (red, *n* = 4) mice and **(E)** total live cells from 3- to 5-week-old Cre^−^ (solid gray, *n* = 3) and L-DKO pTα^−/−^ (red, *n* = 4) mice. PLZF expression in Cre^−^ iNKT cells (purple) is shown as positive control in **(D,E)**. **(F)** Gene expression patterns in L-DKO DP and L-DKO pTα^−/−^ DP cells for genes that are more than twofold upregulated in the latter compared to WT DP cells and are part of the iNKT transcriptional program (Msigdb ID: M18517) or inflammatory responses (Msigdb ID: M5913). **(G)** Enrichment of gene sets in L-DKO pTα^−/−^ DP cells over L-DKO DP cells pertaining to iNKT development and maturation (left), and inflammatory responses (right) using gene set enrichment analysis. Enriched genes from these gene sets are shown in **(F)**. **(H)** Gene expression patterns for genes that are positively (red) or negatively (blue) correlated with Zbtb16 expression. Error bars represent SEM. Statistical significance is represented by *p* values (*<0.05, **<0.005, ***<0.0005, n.s. >0.05).

As expected, L-DKO pTα^−/−^ mice still had a profound block in conventional αβ T cell development due to the lack of pre-TCR signaling (Figures 5A,B). Interestingly, despite the pre-TCR block in L-DKO pTα^−/−^ mice, the deletion of Id proteins seemed to partially rescue the development of DP cells (Figures 5A,C). Upon careful investigation, we found that these DP cells upregulate PLZF (Figure 5D). Our gating strategy excluded iNKT cells recognizing the CD1d tetramer and γδ T cells expressing TCRγδ to ensure that these PLZF^hi^ DP cells are not an artifact of aberrant upregulation of CD4 and CD8 by iNKT or γδNKT cells. Total thymocytes from L-DKO pTα^−/−^ mice also displayed a prevalent innate-like phenotype, as indicated by their PLZF expression pattern (Figure 5E). Thus, blocking conventional αβ T cells with pTα deficiency revealed a pre-TCR independent pathway that drives iNKT and innate-like lineage development in Id2/Id3-deficient mice.

### Initiation of an Innate-Like Transcriptional Program in the Absence of Id Proteins and Pre-TCR Signaling

Our previous data suggested that pre-TCR signaling and Id protein activity is necessary to enforce conventional T cell fate, such that the absence of both gave rise to predominantly innate-like T cell populations in the thymus. We further verified the innate-like phenotype of PLZF^hi^ DP (CD1dTet^−^ TCRγδ^−^ CD4^+^ CD8^+^) cells in L-DKO pTα^−/−^ mice by RNA-Seq analysis. Gene set enrichment analysis (GSEA) verified enrichment of innate-like genes associated with iNKT cell development and inflammatory responses, including *Zbtb16, Gzma*, and *Il2rb*, to be enriched in L-DKO pTα^−/−^ DP cells compared to both L-DKO DP and WT DP cells (Figures 5F,G) (30, 31). Since genes with similar expression patterns can be expected to function together and/or be involved in similar biological processes, we examined expression patterns for genes that correlated positively or negatively with *Zbtb16* across all samples, including WT, pTα^−/−^, L-DKO, and L-DKO pTα^−/−^ DP cells, to discern innate-like genes (Table S1 in Supplementary Material). We found that most genes positively correlated with *Zbtb16* were specifically upregulated in L-DKO pTα^−/−^ DP cells, whereas most genes negatively correlated with *Zbtb16* were downregulated in these cells (Figure 5H). RNA-Seq analysis of the PLZF^hi^ L-DKO pTα^−/−^ DP cells also revealed these cells had undergone TCRα rearrangement with a fairly broad V-J usage (data not shown). These analyses demonstrate the early initiation and adoption of an innate-like transcriptional program specifically in DP cells that arise in the absence of pre-TCR signaling and Id function in the thymus.

### iNKT Cells Are Transcriptionally More Similar to γδNKT Cells than to Their Conventional αβ Counterparts

**Figure 6:**
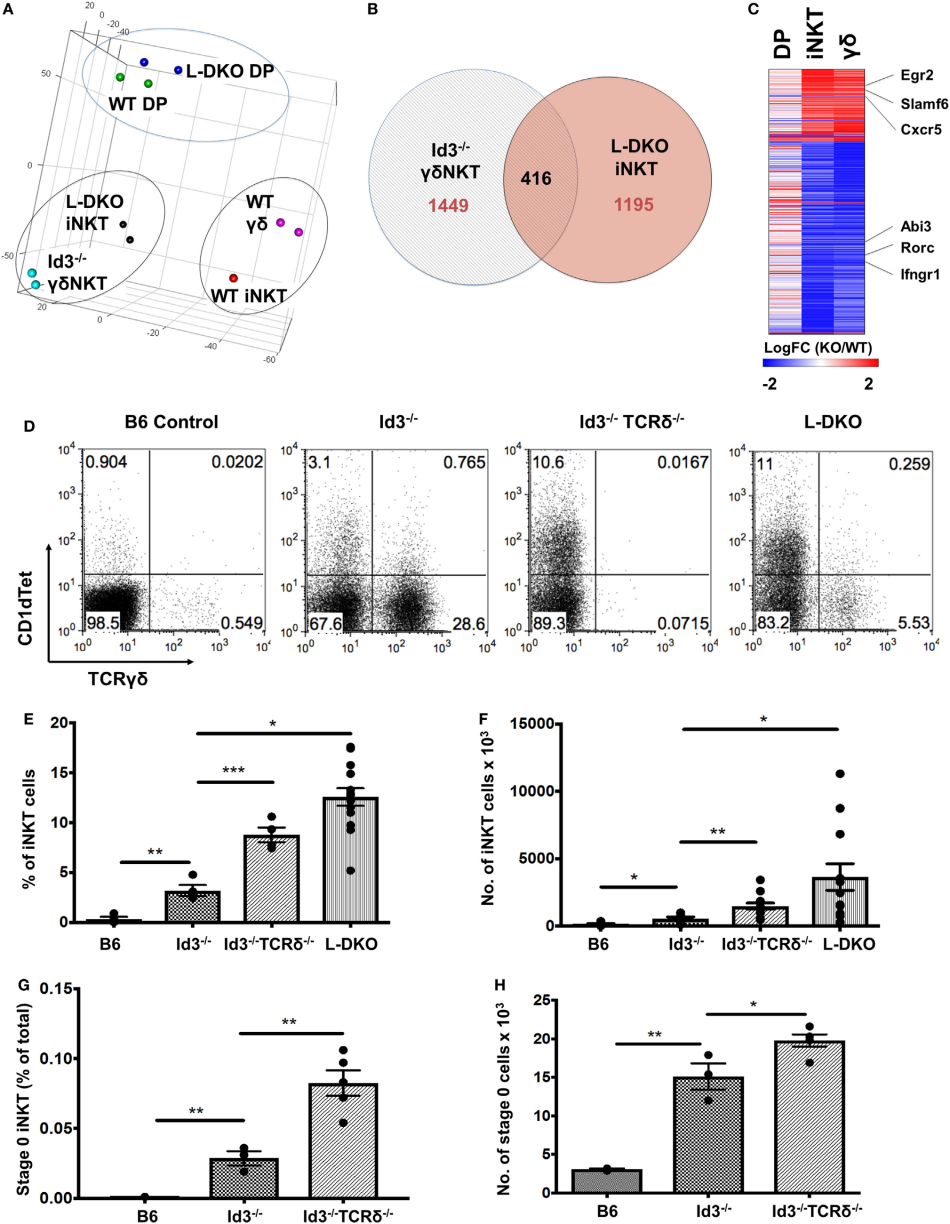
Transcriptional similarity between iNKT and γδNKT cells, whose lineage competition outcome is regulated by Id proteins. **(A)** Principal component analysis of RNA-Seq expression data, with samples grouped according to similarity. **(B)** Genes found to be differentially expressed (fold change >2) in L-DKO iNKT and Id3^−/−^ γδ samples, as compared to their WT counterparts. Numbers indicate unique or shared gene dysregulation between samples. **(C)** Expression patterns of 416 differentially expressed genes identified in **(B)**, in L-DKO DP, L-DKO iNKT, and Id3^−/−^ γδ T cells compared to WT DP, WT iNKT, and WT γδ T cells, respectively. **(D)** Representative distribution of iNKT (CD1dTet^+^) versus γδ T cells in 20-day-old B6 (*n* = 4), Id3^−/−^ (*n* = 4), Id3^−/−^TCRδ^−/−^ (*n* = 4), and L-DKO (*n* = 4) mice. **(E)** Percentage and **(F)** numbers of iNKT cells in 3- to 5-week-old B6 (*n* = 5), Id3^−/−^ (*n* = 7), Id3^−/−^TCRδ^−/−^ (*n* = 13), and L-DKO (*n* = 14) mice. **(G)** Percentage and **(H)** numbers of stage 0 iNKT cells in 2- to 5-week-old B6 (*n* = 3), Id3^−/−^ (*n* = 3), and Id3^−/−^TCRδ^−/−^ (*n* = 5) mice. Error bars represent SEM. Statistical significance is represented by *p* values (*<0.05, **<0.005, ***<0.0005, n.s. >0.05).

In the previous section, our study of L-DKO pTα^−/−^ mice revealed an expansion of both innate-like γδ and iNKT cells. Recent publications have also reported the sharing of transcriptional and effector programs between iNKT cells and γδNKT cells, even though these two lineages are considered to independently diverge at the DP and DN3 stages, respectively (32, 33). Since Id3-deficient (Id3^−/−^) mice support the expansion of innate-like γδNKT cells, we compared WT and Id3^−/−^ γδNKT cells to WT and L-DKO iNKT and DP cells by RNA-Seq (15, 16). Interestingly, both principal component analysis (PCA) and clustering analysis highlighted that iNKT cells are transcriptionally more similar to γδNKT cells than to conventional αβ DP cells, regardless of whether the comparisons were of WT populations or mutant populations (Figure 6A; Figure S3A in Supplementary Material). While there were differences in the gene expression patterns of L-DKO iNKT and Id3^−/−^ γδNKT cells, around 25% of dysregulated genes were similarly affected in both populations (Figures 6B,C). The genes with similar expression patterns, including *Egr2, Slamf6, Rorc*, and *Ifngr1*, were largely specific to the innate-like populations and had distinct expression patterns in L-DKO DP cells (Figure 6C). Given the transcriptional and functional similarities between iNKT and γδNKT cells, and their expansion in Id-deficient mice, we wanted to further examine if Id proteins regulate the lineage competition in these two innate-like populations, by eliminating γδ lineage development and expansion.

We found a significant increase in the iNKT population in Id3^−/−^TCRδ^−/−^ mice as compared to Id3^−/−^ mice (Figures 6D–F). A modest but significant increase was observed in iNKT-committed stage 0 cells, but not in proliferating stage 1 cells (Figures 6G,H). In one study, iNKT cells and γδNKT cells were shown to compete for a thymic niche, based on the reduction in iNKT cells upon expansion of γδNKT cells (34). In contrast, another study has reported that a reduction in iNKT cells does not lead to a corresponding increase in γδNKT cells (35). In order to address this issue, we decided to separate ongoing T cell development from homeostatic expansion associated with void space and examined pre-weaning pups that had not yet undergone full expansion and stabilization of the thymic architecture. A large increase in the iNKT population was observed again in pre-weaning age Id3^−/−^TCRδ^−/−^ mice (Figures S3B,C in Supplementary Material). Age-matched TCRδ^−/−^ mice, which lack total γδ T cells but are wild-type for Id3, did not exhibit a corresponding increase in iNKT cells. These data suggest lineage competition between γδNKT and iNKT lineages in Id-deficient mice.

### E2A Orchestrates a Gene Network that Promotes iNKT and γδNKT Cell Fate in the Absence of Id Proteins

So far, we observed expansion of iNKT, γδNKT, and innate-like DP cells in Id-deficient mice, and our previous results implicated E2A in control of transcription programs that drive iNKT cell development. We wanted to further explore the role of E2A in orchestrating innate-like T cell development. Compared to conventional T cells, however, our understanding of transcriptional programs in innate-like T cells is still in its nascent stages. Currently, PLZF is one of the only well-defined innate-like transcription factors. We, therefore, decided to explore the innate-like transcription program by compiling a reference innate-like gene set from publicly available Immgen data (36).

We hypothesized that genes that are upregulated or downregulated significantly in *both* iNKT and γδNKT cells over other T cell populations in the thymus would be unique to these innate-like lineages and most likely be important for their development and/or function. Our motivation was to delineate genes that are representative of a broad innate-like program, and important for both iNKT and γδNKT cell development, and specifically relevant for stage 0/1 iNKT cells and CD24^hi^ immature γδNKT cells that expand most dramatically in Id-deficient mice (12, 37). Therefore, we compared the gene expression in these WT innate-like T cells against multiple WT conventional T cell populations, including DN3a, DN3b, DN4, DP, post-selection CD4SP, and thymic γδ T cells, as reported in Immgen. This analysis resulted in 189 reference genes, which are significantly overexpressed or repressed in both WT iNKT and γδNKT populations. We added seven other genes to this list that were culled from literature to be important for the development of these populations, but are not significantly overexpressed or repressed in these comparisons (Table S2 in Supplementary Material). It is important to note that our strict criteria would filter out genes important for only one of the populations or for latter stages of maturation and function of either lineage.

**Figure 7:**
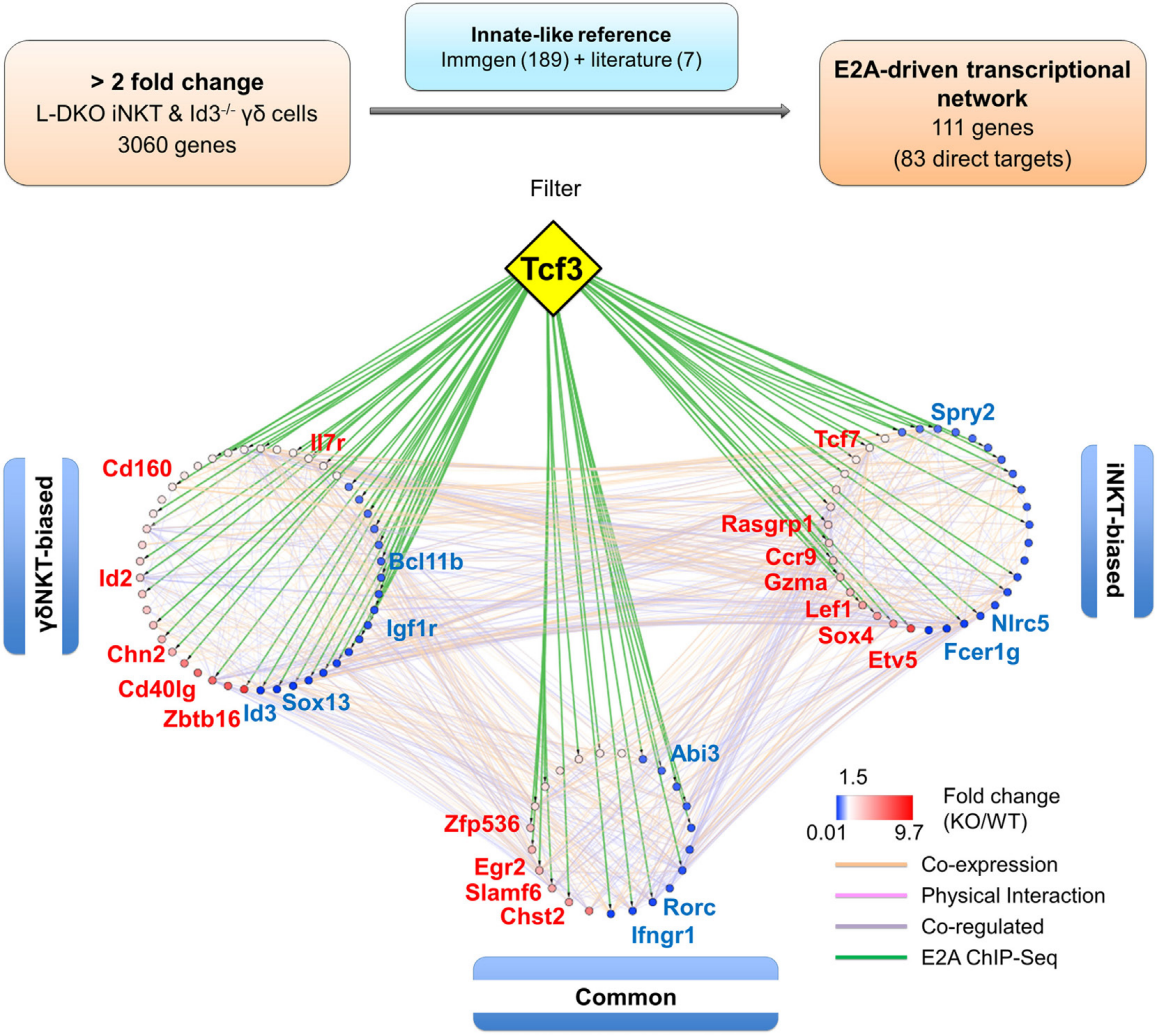
E2A drives a gene network that promotes iNKT and γδNKT fate in the absence of Id proteins. Schematic (top panel) representing the analysis pipeline used to derive the E2A-driven innate-like T cell transcriptional network. Network analysis (lower panel) depicting RNA-Seq expression and E2A ChIP-Seq data for genes part of the innate-like reference gene set. Genes in the “γδNKT-” or “iNKT-biased” groups are colored according to their expression in Id3^−/−^ γδ T cells and L-DKO iNKT cells, respectively. Genes in the “common” group are represented by their average expression in Id3^−/−^ γδ T cells and L-DKO iNKT cells. ChIP-Seq binding of E2A (encoded by Tcf3 gene) to gene targets is represented by green lines. Other interactions between gene targets, classified by GeneMania as co-expression, physical interaction, or co-regulation, are represented by orange, pink, and purple lines, respectively.

When this reference innate-like gene set was compared to the genes identified in our RNA-Seq analysis, we found more than 50% (111 genes) to be dysregulated by at least twofold in either one or both of the cell populations in Id-deficient mice (Figure 7; Table S3 in Supplementary Material). Importantly, E2A directly bound to many (83 of 111 genes) of these differentially expressed reference genes (Table S3 in Supplementary Material). In order to further delineate the role of E proteins in regulating the developmental programs of iNKT and γδNKT cells through these downstream mediators, we divided the 111 genes into three groups based on their expression profiles in L-DKO iNKT and Id3^−/−^ γδNKT cells (Figure S4 in Supplementary Material). Groups 1 and 2 included “biased” genes that were upregulated or downregulated by a significantly larger fold change in one mutant population as compared to the other, i.e., either in L-DKO iNKT or Id3^−/−^ γδNKT cells, compared to their WT counterparts. On the other hand, group 3 included “common” genes that were significantly and similarly upregulated or downregulated in both innate-like populations (Figure S4 in Supplementary Material; Figure 6C). By combining known interactions between these genes with our RNA-Seq and ChIP-Seq data, we created a network map with the three groups of genes demarcated (Figure 7). The distribution of E2A targets across all three groups strongly supported the role of E2A in orchestrating innate-like T cell developmental programs. Our previous observation of diminished iNKT and γδNKT populations in Id2^f/f^Id3^f/f^E2A^f/f^HEB^f/f^ LckCre^+^ (or Q-KO) mice that lack E protein activity further supports the pivotal role of E proteins in the development of these cells (12).

## Discussion

iNKT cells are typically described as a lineage developing in parallel to conventional αβ T cells, bifurcating after TCR-mediated selection at the DP stage. In order to investigate the mechanisms by which Id proteins suppress iNKT development, we studied lineage outcomes and transcriptional programs in Id-deficient mouse models that have a marked expansion of iNKT cells. Our study shows that iNKT lineage development can be distinguished from the conventional αβT lineage as early as the pre-TCR checkpoint, albeit in the context of a unique genetic background. The divergence of iNKT and innate-like T cells from conventional T cells prior to TCR selection has also been proposed in other mouse models with physiological levels of E protein activity. A recent study has used fate-mapping and restriction of Rag2 expression to demonstrate an alternate developmental pathway for a subset of the iNKT population, such that these cells do not arise from the conventional DP stage (38). In line with this, we uncovered a pre-TCR independent pathway for the development of iNKT cells using L-DKO pTα^−/−^ mice. It is likely that the depletion of Id proteins unleashes the “early,” pre-TCR-independent developmental program for iNKT and other innate-like T cells, which otherwise occurs at much lower frequencies on a wild-type genetic background. Consequently, we have also observed heterogeneous innate-like αβ T cell lymphomas derived from iNKT, CD1dTet^−^, or T_FH_ cells in Id2/Id3-deficient mice (14, 39). Cumulatively, our findings support a layered (21), rather than a parallel developmental structure that coordinates the distinct fates of iNKT and conventional αβT cells during T cell development in the thymus.

The loss of iNKT cells in L-DKO CD1d^−/−^ mice emphasizes the critical role of the selection step in iNKT cell development (39). However, TCRα repertoire sequencing of preselection DP cells from these mice demonstrated an increased frequency of Vα14-Jα18 rearrangements, suggesting that the lack of Id proteins can promote iNKT-specific rearrangements prior to, and independent of their selection. It remains to be determined if E2A can regulate the timing or outcomes of TCRα rearrangement to favor expression of the iNKT TCR. The combined genome-wide binding and transcriptional data revealed E2A-mediated transcription programs that support the development of γδNKT and iNKT lineages, providing a direct explanation to the several previous reports of expansion of iNKT and innate-like populations in the absence of Id proteins. This analysis identified E2A as an upstream regulator of genes critical for iNKT and γδNKT lineage differentiation, including *Zbtb16, Slamf6*, and *Egr2* (40–42). Genes that are associated with iNKT1 and iNKT17 cytokine profiles, such as *Ifngr1* and *Rorc*, were found to be significantly downregulated in both Id3^−/−^ γδNKT and L-DKO iNKT cells, supporting the involvement of E2A in preferentially driving iNKT2 and γδNKT lineage development in the absence of Id proteins. Our data also serves as a framework and repository to add new genes as they are increasingly identified by our group and others to be important for innate-like T cell development and effector functions.

Although innate-like T cells represent only a small fraction of the thymic population, their indispensable roles in mounting rapid immune responses in different contexts warrants a holistic understanding of the regulation of their concurrent development with conventional T cells in the thymus. Here, we characterized E2A-driven transcription programs that promote innate-like T cell development prior to TCR selection and independent of pre-TCR signaling, which are otherwise suppressed by Id proteins. Not surprisingly, phylogenetic analysis of innate-like T cells and their associated transcription factors indicates that these cells emerged much earlier than conventional T cells in the course of evolution (21, 43). Hence, we propose that innate-like lineage specification precedes conventional αβ T cells in the thymus and that evolutionary pressures necessitated Id-mediated suppression to ensure the predominance of conventional αβ T cells. Our data also suggest that Id proteins are potent suppressors of iNKT cell fate at the pre-TCR checkpoint.

## Materials and Methods

### Mice

Id2^f/f^Id3^f/f^LckCre^+^ (L-DKO), Id3^−/−^, and Id3^−/−^ TCRδ^−/−^ mice were generated as previously described (12, 44). CD1d^−/−^ mice were purchased from Jackson Laboratory (Strain 008881) and bred with L-DKO mice to generate L-DKO CD1d^−/−^ mice. L-DKO pTα^−/−^ mice were generated by breeding L-DKO mice with pTα^−/−^ mice (28), which were a generous gift from David L. Weist (Fox Chase Cancer Center, Philadelphia, PA, USA). All mice were bred in a specific pathogen-free facility of Duke University Division of Laboratory Animal Resources, and all procedures were performed according to protocols approved by the Institutional Animal Care and Use Committee.

### Cell Sorting and RNA Extraction

All cells were sorted in FACS buffer using a MoFlo XDP cell sorter. Total mRNA from sorted cells was extracted using an RNAqueous Kit (Life Technology) according to manufacturer’s protocol.

### ChIP-Seq Analysis

26 × 10^6^ iNKT and 30 × 10^6^ DP cells were sorted and pooled from multiple L-DKO mice for the E2A ChiP-Seq analysis. iNKT (CD1dTet^+^ TCRβ^+^) and DP (CD1dTet^−^ CD4^+^ CD8^+^) cells were sorted from 3- to 5-week-old L-DKO mice. Cells were fixed with 1% formaldehyde and 1.5 mM EGS [ethylene glycol-bis(succinic acid N-hydroxysuccinimide ester)]. Crosslinked cells were lysed, nuclei were extracted, and sonicated using Bioruptor Plus (Diagenode) and immunoprecipitated with E2A (V-18, Santa Cruz Biotechnology, Lot G0814) antibody. After elution and reverse crosslinking, RNA and proteins were digested, followed by DNA purification using a ChIP DNA Clean and Concentrator kit (Zymoresearch). Libraries were prepared with the NEBNext primer set, which included applying ChIP DNA to end repair, A-tailing, adapter ligation, and PCR amplification. Samples were cleaned and size selected by 8% PAGE or AMPure beads (Agencourt). Sequencing was done on HiSeq4000 platform (Illumina).

ChIP-Seq sequenced reads were aligned to the mm9 genome using Bowtie (45) software (version 1.1.2, parameters: -chunkmbs 128 -mm -m1 -best -strata -p4 -S -q). Peaks were called using MACS (46) (version 1.4.2, default parameters). Peaks were then annotated by the NGS: Peak Annotation tool on Nebula (47). Bed and wiggle files were generated by MACS for visualization using the Integrative Genomic Viewer (48). *De novo* motif analysis was done using the findmotifs.pl program with HOMER (49) (v4.7.2, 50, or 200 bp within each peak).

### RNA-Seq Analysis

iNKT (CD1dTet^+^ TCRβ^+^), γδNKT (TCRγδ^+^ CD3^+^), and DP (TCRγδ^−^ CD4^+^ CD8^+^) cells were sorted from 4- to 5-week-old WT (total 2 × 10^5^ γδ T, 2 × 10^5^ iNKT cells, 2 × 10^6^ DP cells), Id3^−/−^ (total 15 × 10^6^ γδ T cells), or L-DKO (total 15 × 10^6^ iNKT cells, 15 × 10^6^ DP cells) mice. After RNA extraction using the RNAqueous kit, and quality was assessed using the Agilent Bioanalyzer RNA Pico chip. Ribosomal RNA was depleted using the RiboErase method from Kapa Biosystems. In short, 1 μg of total RNA was hybridized with 1 μg of hybridization oligos tiling the 18s, 28s, 5.8s, and mitochondrial rRNA sequences. Each sample was then RNaseH treated to degrade complementary rRNA sequence. The product was cleaned and purified using 2.2× AMPure beads (Agencourt). The cleaned product was DNase treated to degrade the DNA oligo mix. The remaining rRNA depleted samples were then purified using 2.2× AMPure XP beads. The Kapa Stranded RNA-Seq Kit was used to generate stranded Illumina sequencing libraries (Kapa Biosystems). RNA from was fragmented at 94°C for 6 min. Briefly, RNA was hybridized to random primers, followed by first-strand cDNA synthesis, second-strand cDNA synthesis with marking, A-tailing, ligation of Illumina paired-end adapters with 8 bp barcodes, and nine cycles of PCR amplification. Reactions were purified with Agencourt AMPure XP beads where necessary. Libraries were multiplexed in equimolar amounts, and sequenced as paired-end 50-bp reads using a HiSeq2500 platform (Illumina).

A second, independent round of RNA-Seq was done with DP (TCRγδ^−^ CD4^+^ CD8^+^) cells sorted from 5 weeks old WT (9 × 10^5^ cells), pTαKO (4.5 × 10^5^ cells), L-DKO (9 × 10^5^ cells), and L-DKO pTαKO (9 × 10^5^ cells) mice. 150 bp paired-end sequencing was done on the HiSeq2500 platform (Illumina).

RNA-Seq sequencing reads were first trimmed using Trimmomatic (50). Read alignment was done using Tophat and expression quantification was done using Cufflinks (51). Log2-transformed FPKM (fragments per kilobase exon-model per million reads mapped) were used for downstream analyses. Further filtering of low quality genes, PCA, statistical analysis, and visualizations were done using R (52). Pathway analysis was done using the Molecular Signatures Database (MSigDB) v5.2 (53).

### TCRα Repertoire Sequencing

Preselection DP (CD4^+^ CD8^+^ CD69^−^) cells were sorted from 3- to 4-week-old CD1d^−/−^ or TKO mice. RNA was extracted from sorted cells, and reverse transcribed into cDNA by murine leukemia virus reverse transcriptase (Life Technology). Sequences specific for Vα8^+^ and Vα14^+^ cells were isolated and amplified using nested PCR with Vα-specific and Cα primers, followed by indexed Vα primers. Barcoded sequences were finally amplified with common adapter-specific primers, gel purified, and sequenced using Ion Torrent technology (Applied Biosystems). All TCR repertoire analysis was done using IMGT HighV-QUEST and its statistical tool with default parameters (54).

### Flow Cytometry

Surface marker antibodies were used according to manufacturer’s protocol (Biolegend). Intracellular staining with PLZF antibody (eBioscience) was done using the Foxp3 staining buffer kit (eBioscience). CD1d tetramers were received from the Tetramer Facility of the National Institutes of Health. Stained samples were run on a FACSCanto II machine (BD Biosciences) and data was further analyzed with FlowJo software (Tree Star). Bar graphs were drawn using GraphPad Prism (GraphPad Software). Two-tailed student’s *t*-test was used for statistics, with *p*-values less than 0.05 considered significant.

### Innate Gene Signature and Network Analysis

Raw microarray expression data was requested and downloaded from Immgen for selected subsets: preT_DN3A_Th (DN3a), preT_DN3B_Th (DN3b), T_DN4_Th (DN4), T_DP_Th (DP), T_4SP69+_Th (post-selection CD4SP), NKT_44-NK1_1-_Th (stage 0 and 1 iNKT cells), Tgd_Th (total thymic γδ T cells), Tgd_vg1 + vd6 + 24ahi_Th (immature Vγ1.1Vδ6.3 cells). Average gene expression among DN3a, DN3b, DN4, DP, and CD4SP cells was assumed to be the reference conventional αβ T cell population. Total thymic γδ T cells were considered as reference for conventional γδ T cell population. Fold change in expression for iNKT and γδNKT cells was calculated with respect to the reference conventional αβ and γδ T cell populations, respectively. Genes that had more than 1.5-fold upregulation or 0.6-fold downregulation among both iNKT and γδNKT cells were considered to represent the “innate-like gene signature.” These moderately relaxed fold change parameters allowed us to ensure that maximal numbers of appropriate genes were captured in this analysis. 189 genes were, therefore, identified from these specific expression patterns among WT iNKT and γδNKT cells. Additionally, we also included seven other genes—*Tcf3* (E2A), *Id2, Id3, Lef1, Sox13, Blk*, and *Sox4—*which have been reported to play important roles in iNKT and γδNKT lineage development, but did not have expression patterns that fit our criteria, i.e., being significantly upregulated or downregulated in both cell types as compared to reference populations. The total 197 genes constituted our innate-like gene signature, derived from Immgen and literature.

111 of the 197 signature genes were found to be dysregulated in Id3^−/−^ γδ and/or in L-DKO iNKT cells. Other known interactions between these 111 genes were retrieved from GeneMania (55). 83 of the 111 genes were also identified as E2A targets, which had E2A binding to the enhancer, promoter, intragenic, intergenic, or downstream regions of these genes, as annotated by Nebula. These interactions, ChIP-Seq targets, and gene expression patterns of the 111 genes were represented as a network using Cytoscape3.4.0 (56).

### Gene Set Enrichment Analysis

The GSEA (53, 57) desktop application (v2.0) was used to analyze the log2FPKM expression patterns in L-DKO and L-DKO pTα DP samples. 9,245 genes that were unchanged in L-DKO DP samples as compared to WT DP samples were included in this analysis. Enrichment in L-DKO pTα DP samples over L-DKO samples was determined using weighted, log2 (ratio of classes) parameters and 1,000 permutations. The iNKT development and maturation gene set (31) (Msigdb gene set M18517) and inflammatory responses gene sets (Msigdb gene set M5932) were downloaded from Msigdb and used as is.

### Correlation Analysis

To determine correlation with Zbtb16 expression, Pearson and Spearman correlation coefficients were determined for all genes across six samples, including replicates of WT DP, pTαKO DP, L-DKO pTαKO DP, and L-DKO DP, as derived from RNA-Seq analysis. Genes with both coefficients greater than or equal to 0.7 were considered to be positively correlated, and those with both coefficients less than or equal to −0.7 were considered to be negatively correlated with Zbtb16 expression. Scatter plots were generated using a custom R script.

### Data and Materials Availability

Complete E2A ChIP-Seq and RNA-Seq data can be accessed from NCBI GEO using the following link: https://www.ncbi.nlm.nih.gov/geo/query/acc.cgi?token=ebgrcmaefjmzlmr&acc=GSE89849.

## Ethics Statement

All mice were bred in a specific pathogen-free facility of Duke University Division of Laboratory Animal Resources, and all procedures were performed according to protocols approved by the Institutional Animal Care and Use Committee.

## Author Contributions

Conceptualization and writing—original draft: SR and YZ. Methodology: SR, AM, and YZ. Software: SR, AR, LL, and DR. Investigation: SR, AM, CL, and YZ. Formal analysis: SR. Writing—review and editing: SR, AM, CM, SD, and YZ. Funding acquisition: CM and YZ. Resources and supervision: SD, CM, and YZ.

## Conflict of Interest Statement

The authors declare that the research was conducted in the absence of any commercial or financial relationships that could be construed as a potential conflict of interest.
