# Supplementary figures and images for: Id Proteins Suppress E2A-Driven Invariant Natural Killer T Cell Development prior to TCR Selection
Source: Front Immunol. 2018 Jan 24;9:42. doi: 10.3389/fimmu.2018.00042 (PMC5787561; doi:10.3389/fimmu.2018.00042)

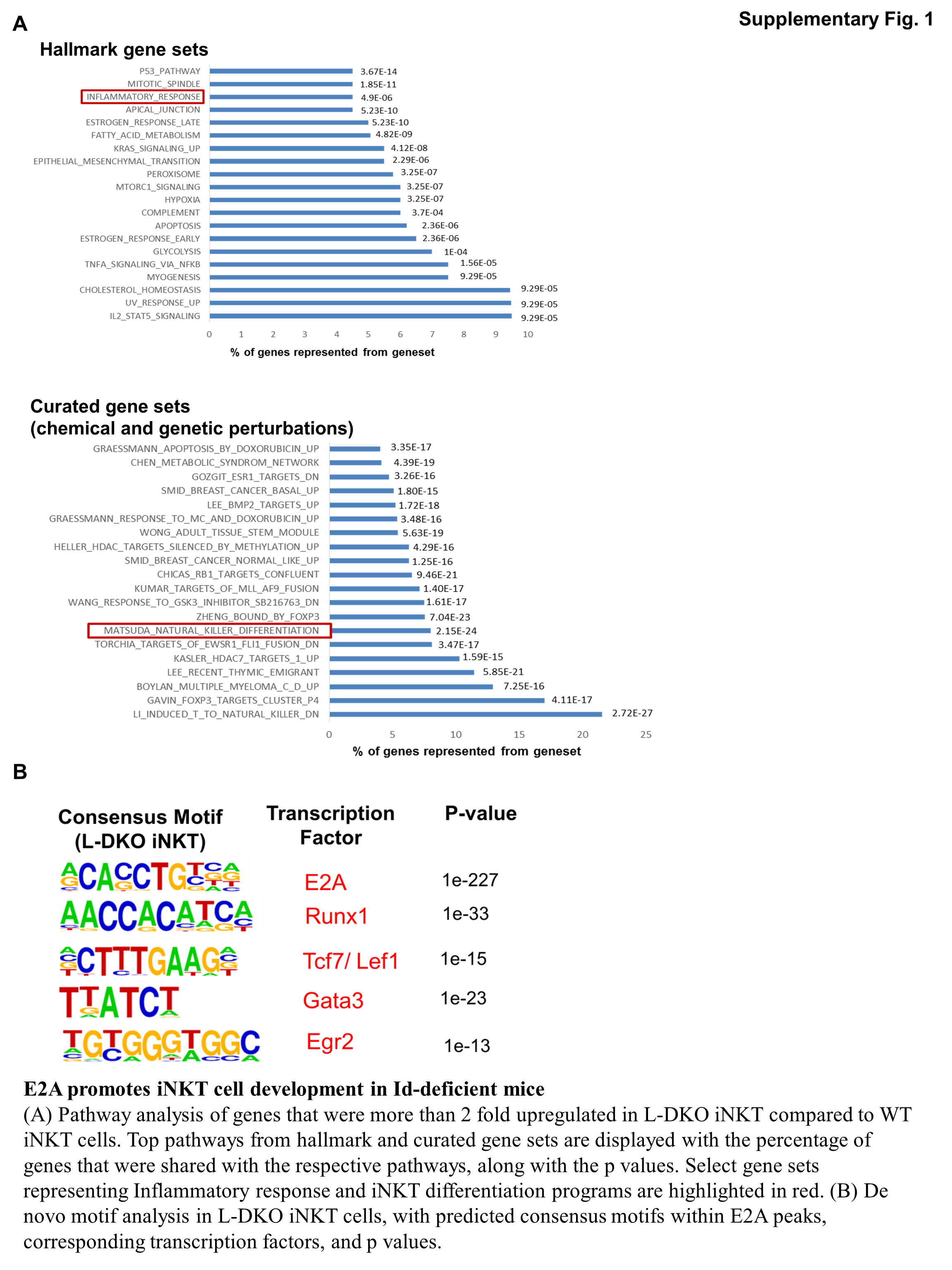

Supplement: Supplementary file 4 [file Image_1.tif]

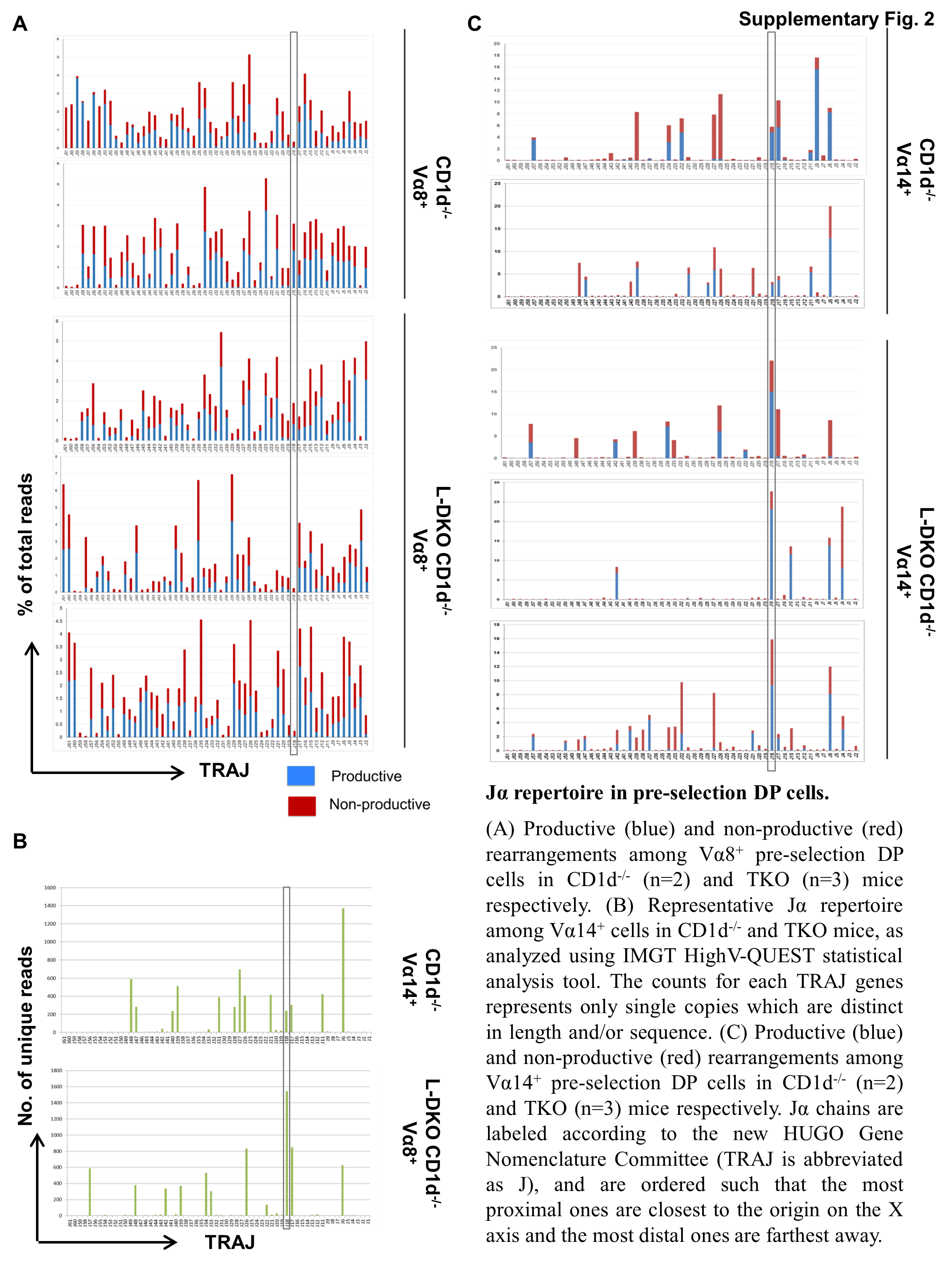

Supplement: Supplementary file 5 [file Image_2.tif]

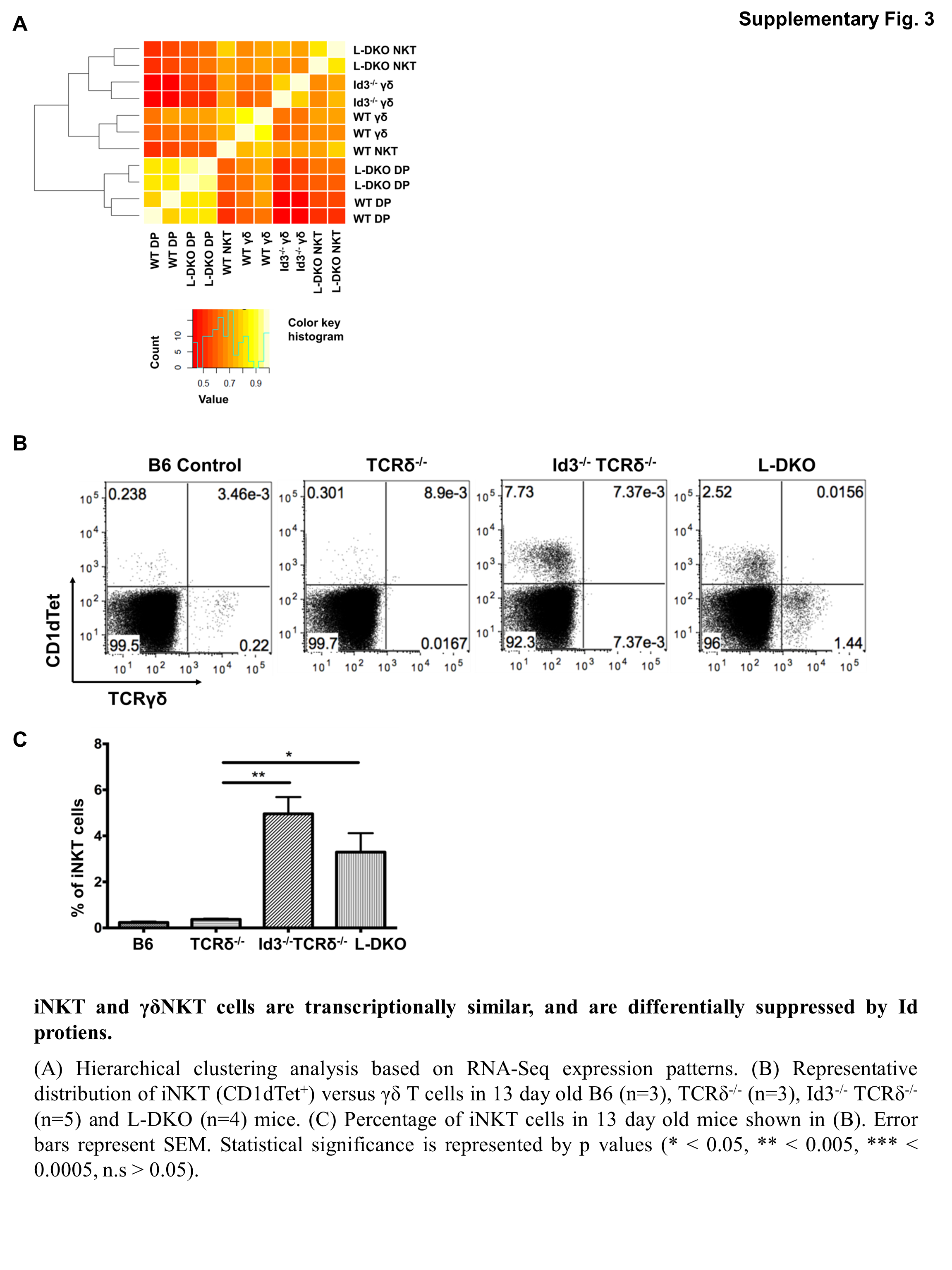

Supplement: Supplementary file 6 [file Image_3.tif]

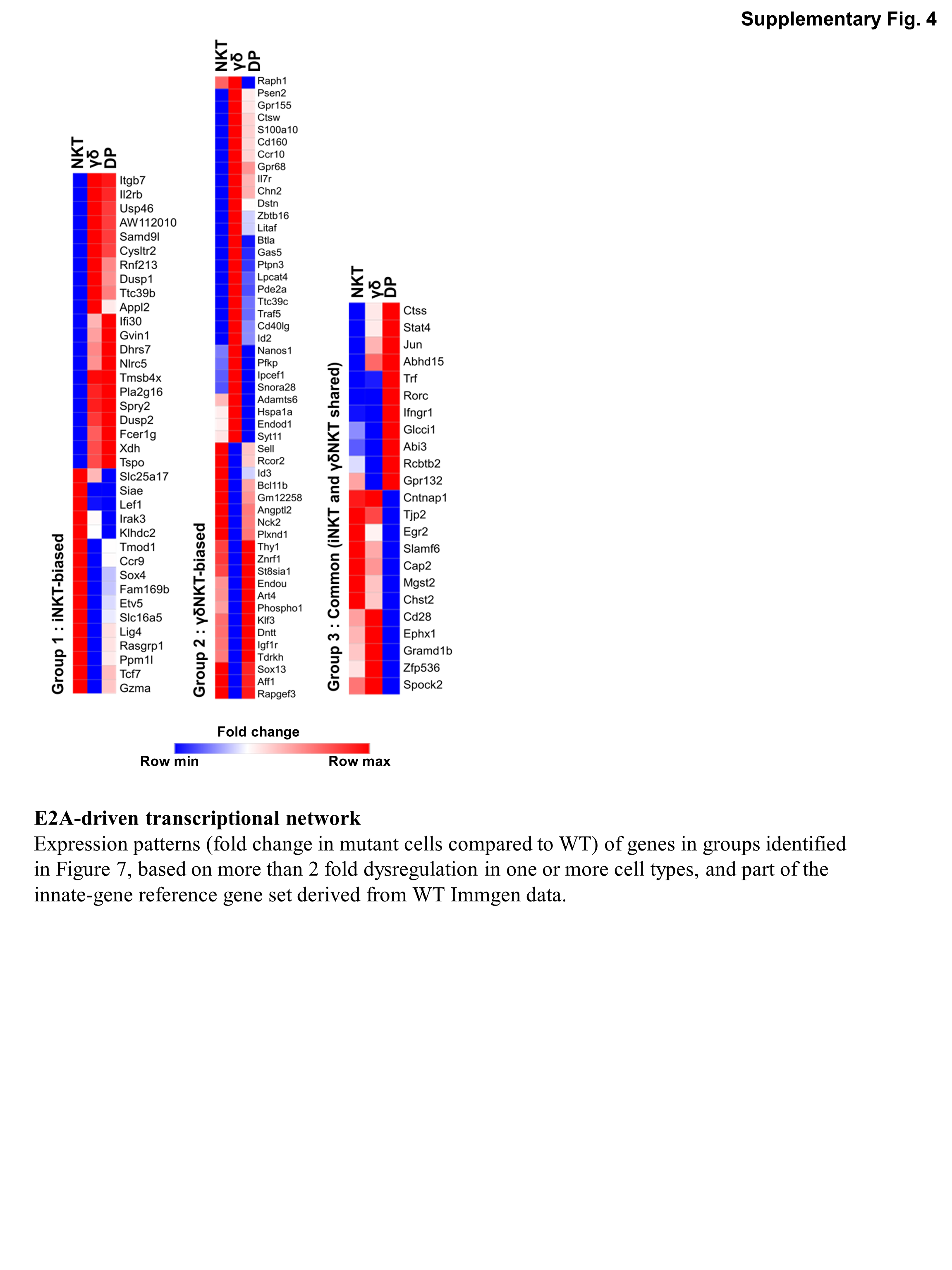

Supplement: Supplementary file 7 [file Image_4.tif]
